# Neurofunctional Correlates of Emotional Dysregulation: Systematic Review and ALE Meta‐Analysis

**DOI:** 10.1002/brb3.71376

**Published:** 2026-04-16

**Authors:** Ern Wong, Riccardo Loconte, Francesca Terigi, Elisa Giani, Matteo Bucci, Maurilio Menduni De Rossi, Davide Coraci, Annarita Milone, Gabriele Masi, Luca Cecchetti, Gianluca Sesso

**Affiliations:** ^1^ IMT School for Advanced Studies Lucca Italy; ^2^ University of Pisa Pisa Italy; ^3^ Sant'Anna School of Advanced Studies Pisa Italy; ^4^ Developmental Psychiatry and Psychopharmacology Unit IRCCS Stella Maris Foundation Pisa Italy; ^5^ Social and Affective Neuroscience (SANe) Group, MoMiLab Research Unit IMT School for Advanced Studies Lucca Lucca Italy

**Keywords:** activation likelihood estimation (ALE) meta‐analysis, emotional dysregulation, fMRI, systematic review

## Abstract

**Background:**

Emotional dysregulation (ED) is a transdiagnostic feature of several psychiatric and neurodevelopmental disorders, characterized by heightened emotional reactivity, mood instability, and difficulties regulating emotional responses.

**Methods:**

In this study, an activation likelihood estimation (ALE) meta‐analysis was conducted to examine the neural underpinnings of ED across different clinical populations. A systematic search based on preferred reporting items for systematic reviews and meta‐analyses (PRISMA) guidelines identified 35 task‐based fMRI studies (*n* = 1989 subjects), including patients with borderline personality disorder (BPD), attention‐deficit/hyperactivity disorder (ADHD), bipolar disorder (BD), and other conditions.

**Results:**

Hyper‐activation was observed in emotion‐related regions, particularly bilateral amygdala and left insula, indicating heightened emotional sensitivity and reactivity. Hypo‐activation, detected through Bayesian thresholding, was found in areas such as the anterior cingulate cortex and supplementary motor area, suggesting impairments in cognitive control and emotional regulation. Functional connectivity analysis revealed distinct patterns of coactivation, with the amygdala showing isolated activity and the left insula coactivating with regions related to sensory processing and cognitive control.

**Conclusions:**

These findings provide new insights into the neural circuitry of transdiagnostic ED and suggest that disorders, such as BPD, ADHD, and BD, share common neural mechanisms, particularly in regions involved in emotional reactivity and cognitive regulation. The results have important clinical implications for developing targeted interventions to address both emotional and cognitive deficits in ED. Future research should explore causal mechanisms and incorporate diverse clinical populations to further understand neurobiological basis of ED.

## 1 Introduction

People deal with emotions every day, as they are exposed to a wide range of emotionally arousing situations throughout their daily lives. Sometimes emotions can be troublesome and must be managed somehow. From childhood, individuals develop emotion regulation (ER) strategies to manage intense feelings, adapt behavior, and maintain personal and social well‐being (Gross and Jazaieri 2014). However, not everyone develops effective regulation skills. Some struggle to control their emotions, leading to difficulties in functioning (Shaw 2014). Emotional dysregulation (ED) is a common issue in psychiatric care, yet its definition varies across the literature (Marwaha et al. 2014). Most researchers agree that ED is linked to rapid shifts in mood, inappropriate emotional responses, and extreme reactions to social cues (Broome et al. 2015). ED overlaps with terms like emotional instability and mood swings, further complicating its characterization in psychiatric disorders (Perugi et al. 2015).

Clinically, ED appears as hyperarousal, lability, mood shifts, and low tolerance to frustration (Masi et al. 2021). In youth, irritability is a common subtype of ED, often featuring reactive aggression and temper outbursts (Leibenluft and Kircanski 2021). Various measures have been developed to assess ED in youth (Freitag et al. 2023; Sesso et al. 2021). ED is especially prevalent in psychiatric populations, such as those with attention‐deficit/hyperactivity disorder (ADHD), where severe irritability is experienced in up to 50%, and ED has been suggested as a core feature of the disorder (Shaw et al. 2014). ED is also central to clinical conditions, such as borderline personality disorder (BPD) and disruptive mood dysregulation disorder (DMDD) (American Psychiatric Association 2022), as well as severe mood dysregulation (Brotman et al. 2006).

Despite challenges in defining ED, it is recognized as a multifaceted, transdiagnostic issue across psychiatric and neurodevelopmental conditions (Carmassi et al. 2022; Masi et al. 2021). This aligns with the Research Domain Criteria (RDoC) framework, which examines mental health through dimensions of behavior that cut across diagnostic boundaries (Insel et al. 2010). Studies using the RDoC or similar transdiagnostic approaches have explored the neural basis of ED. For instance, previous meta‐analyses combining patterns of activation across different conditions were aimed at identifying neurofunctional correlates of this construct. Schulze et al. (2019) found hyper‐activation in the right medial cingulate gyrus and hypo‐activation in frontal and occipital areas in patients with affective disorders. Similarly, McTeague et al. (2020) showed increased activation in the amygdala and hippocampus and reduced activation in prefrontal areas during emotional processing, highlighting widespread neural disruptions in ER across psychiatric disorders. On the other hand, two other meta‐analysis groups led by Khodadadifar et al. (2022) and Lee et al. (2023), respectively, found limited neural convergence, suggesting that neural mechanisms of ED are complex and not yet fully understood.

The primary limitation of these previous meta‐analyses is that they all have focused on specific age ranges within their samples (children vs. adults vs. elderly), specific tasks (e.g., ER strategies), or specific conceptualizations of ED (e.g., irritability). In light of this and other limitations, our activation likelihood estimation (ALE) meta‐analysis aims at identifying the neural underpinnings of ED as a transdiagnostic phenomenon, across diverse clinical populations and diagnostic categories, by applying both a canonical ALE approach and a Bayesian thresholding method.

## 2 Materials and Methods

### 2.1 Search Strategy

**FIGURE 1:**
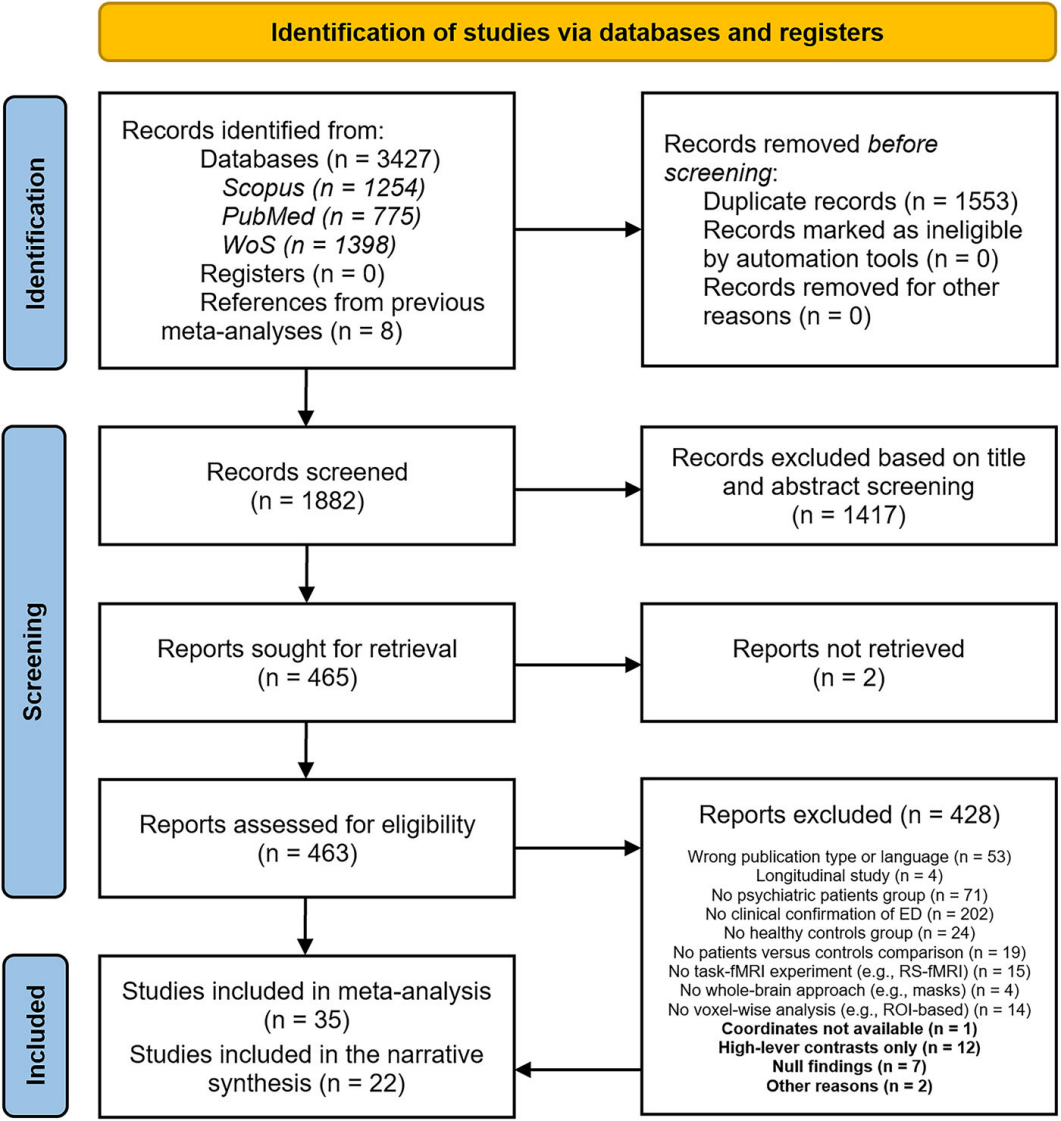
PRISMA flowchart.

A systematic search was conducted on the basis of the PRISMA (preferred reporting items for systematic reviews and meta‐analyses) guidelines (Page et al. 2021) to identify task fMRI studies for the purpose of this review and meta‐analysis. The current work was preregistered on PROSPERO (CRD42022331803). The systematic search was run in three different databases, namely, Scopus, PubMed, and Web of Science. To ensure that the search included all the key fMRI studies of interest, the identified list of articles was cross‐checked with the reference lists of four recent systematic reviews and meta‐analyses retrieved through our search, respectively (Khodadadifar et al. 2022; Lee et al. 2023; McTeague et al. 2020; Schulze et al. 2019). Details of the screening procedures and information regarding the exclusion of selected articles are outlined in the PRISMA flowchart (Figure 1).

In this study, ED was conceptualized using a transdiagnostic approach, imposing no restrictions on the demographic factors associated with the samples recruited in the studies, on the diagnostic categories used for clinical evaluation, and on the type of ED assessment. Such conceptualization, hence, gave rise to the following search terms and their derivatives:

‐ A: Emotion* OR Affect* OR Mood
‐ B: Dysregulat* OR Lability OR Instab* OR Swing*
‐ C: Irritab*
‐ D: fMRI OR Functional Magnetic Resonance OR Functional MRI

and the following search string (((A and B) or C) and D), which was used to search for peer‐reviewed fMRI articles published in English, until November 20, 2023.

### 2.2 Eligibility Criteria

Studies were first conservatively screened solely based on title and abstract upon the following criteria: (a) cross‐sectional studies conducted on (b) a cohort of clinical patients versus (c) healthy controls (HCs) with (d) participants undergoing a task‐fMRI experiment. Then, full‐text articles were screened to assess their eligibility for our meta‐analysis based on the following criteria:

- Publication type: Original articles published in English presenting experimental studies with cross‐sectional design (i.e., longitudinal studies were not included); reviews were retrieved for reference list screening and theoretical purposes but finally discarded;
- Participants: Patients who received (1) a diagnostic confirmation of any psychiatric and/or neurodevelopmental disorder as confirmed by ICD/DSM‐based criteria *and* (2) a clinical confirmation of ED based on a significantly higher level of symptoms score on structured or unstructured clinical interviews or questionnaires compared to a control group (see the full list of acceptable ED measures included in the Supporting Information) as well as based on standardized diagnostic criteria of specific psychiatric conditions (i.e., DMDD, SMD, and BPD with explicitly confirmed criteria 6 and/or 8);
- Controls: Comparison group of HCs with the clinically confirmed absence of any psychiatric or neurodevelopmental disorder;
- Comparison: A direct comparison between patients with ED and HCs;
- Experiment: Task‐based fMRI experiment with any type of task and condition (i.e., resting state‐fMRI and PET studies were excluded);
- Analysis: (1) Whole‐brain approach (i.e., studies applying brain masks were excluded) with (2) voxel‐wise analysis (i.e., region of interest (ROI)‐based studies were not included);
- Outcomes: (1) Coordinates of peak activations should be available in the main text of the article, in the Supporting Information, or upon request to authors; (2) low‐level contrasts only were included (i.e., higher level interactions among covariates were not considered); (3) only significant results could be analyzed (i.e., null findings were to be excluded). A narrative synthesis of studies excluded for these latter reasons was noteworthily reported.

Given the abovementioned variability in operational definitions and assessment tools across studies, ED was defined dimensionally rather than categorically by targeting a shared lower order affective control dimension cutting across diagnostic categories. Specifically, eligible studies were required to assess a construct explicitly reflecting core ED features, including heightened emotional reactivity, affective instability and lability, chronic irritability, and impaired modulation of emotional responses. Although clinical measures differed across selected studies, inclusion was restricted to constructs capturing this shared functional definition. This approach aligns with a transdiagnostic framework proposed by the RDoC, which prioritizes common behavioral dimensions over diagnostic labels.

### 2.3 Screening Procedure and Data Extraction

Seven researchers (E.W., R.L., F.T., E.G., M.B., M.M.D., and G.S.) independently screened one‐seventh of titles and abstracts of the retrieved articles to identify relevant publications. If articles appeared potentially eligible, but no abstract was available, or some of the eligibility criteria were not fully detailed in the abstract, the full‐text article was retrieved. Any controversial aspect found in the screening process was discussed among the researchers and resolved by consensus. On the basis of full‐text articles, data were extracted by the authors on study design, summary measures about participants, clinical measurements, and outcomes, using a standardized data extraction form. Any uncertainties were discussed among the researchers and resolved by consensus. Coordinates reported in the Montreal Neurological Institute (MNI) space were then extracted. Coordinates reported in Talairach's stereotactic space were converted into MNI space through Brett's algorithm implemented in GingerALE software (version 3.0.2, https://www.brainmap.org/ale/). To ascertain the presence of consistent patterns of fMRI hypo/hyper‐activations, as well as to determine the force of evidence of our findings, we used both Bayesian‐based and canonical versions of the ALE method.

### 2.4 Canonical ALE

The coordinate‐based meta‐analysis was conducted using the ALE algorithm as implemented in GingerALE. This method tests for spatial convergence across studies by modeling a 3D Gaussian probability distribution over the reported foci, estimating the likelihood of true activation. The width of these probability distributions is based on empirical estimates of spatial uncertainty in neuroimaging results, which depend on factors, such as sample size and variability across templates. Larger sample sizes are assumed to provide more reliable estimates of actual activation, leading to narrower distributions.

Spatial probabilities were combined to create modeled activation (MA) maps for each experiment, derived by taking the maximum probability associated with each focus. The final ALE image was generated by merging the MA maps from all studies. ALE scores were compared against a null distribution representing random spatial associations expected by chance to differentiate true signal from noise. Following the recommendations by Eickhoff et al. (2016) and Müller et al. (2018), the ALE results were corrected for multiple comparisons using family‐wise error correction (FWE‐c) with a cluster‐level inference threshold of *p* < 0.05. The voxel‐level cluster‐forming threshold was set at *p* < 0.001, using 1000 permutation runs, and clusters were required to be ≥200 mm^3^ in size.

### 2.5 ALE With Bayesian Thresholding

To further improve the inferences that could be drawn from our meta‐analysis, we complemented the canonical ALE approach with the minimum Bayes Factor (mBF) thresholding method described by Costa and colleagues (Costa et al. 2023). This latter does not require a minimum number of studies to produce unbiased results (Costa et al. 2023) and offers a way to measure the strength of evidence on a continuum that would be binarized in the canonical frequentist ALE approach. Briefly, the hypothesis of our effects of interests was modeled as a Gaussian distribution. Using Z‐maps obtained from GingerALE, mBF maps were subsequently computed by applying the exponentiation below:

(1) $$\mathrm{mBF}=e^{-\frac{z^{2}}{2}}\;$$

As this represents the evidence of the null hypothesis against the alternative, we take the reciprocal to obtain the evidence of the alternative for the sake of interpretation. Following the distribution of the evidence categories for the Bayes Factor proposed by Kass and Raftery (1995), the mBF‐ALE threshold was set at “very strong evidence” (i.e., BF ≥150), with a cluster size ≥200 mm^3^. This analysis was conducted using a Python implementation (https://github.com/ErnWg/minBayes_ALE.git) of the algorithm (Costa et al. 2023).

### 2.6 Meta‐Analytic Connectivity Modeling (MACM) Analysis

MACM was conducted using Sleuth v3.0.4 and GingerALE v3.0.2, with significant clusters from the ALE analysis serving as seed regions. For each seed, the BrainMap database was searched for experiments on healthy participants that reported at least one activation focus within the seed region. An ALE analysis was then performed on the extracted experiments to assess spatial convergence across the reported foci. The statistical thresholds used were consistent with those applied in the ALE analysis (cluster‐forming threshold *p* = 0.001; cluster‐level FWE correction *p* = 0.05, 1000 permutations). High convergence within the seed region would indicate strong activation overlap, whereas convergence outside the seed region would suggest other brain areas consistently co‐activated with the seed, reflecting task‐based functional connectivity (Kotkowski et al. 2018; Robinson et al.^2012^). Separate searches were conducted in Sleuth for each identified peak ROI. The search criteria included the following: (1) activations only, (2) context set to “Normal Mapping,” (3) subject diagnosis limited to HCs, and (4) each ROI as defined in MNI space from the canonical ALE results. Studies matching these criteria were then imported into Sleuth workspace.

### 2.7 Functional Decoding With NeuroSynth

Last, functional decoding of activation patterns identified by means of ALE meta‐analysis was conducted with NeuroSynth software. NeuroSynth (Yarkoni et al. 2011) is a software for large‐scale, automated analyses of over 14,000 published fMRI studies. Particularly, it allows decoding whole‐brain activation maps by identifying terms—as proxies of cognitive processes, psychological states, and neuroanatomical regions investigated in studies—that correlate most strongly with the input activation map. In this study, Core NeuroSynth Tools (available at https://github.com/neurosynth/neurosynth.git; see Supporting Information—NeuroSynth for the specific code used for offline analysis) were employed to decode ALE‐based maps of hyper‐ and hypo‐activations, providing additional support for the interpretation of the meta‐analysis results.

## 3 Results

### 3.1 Study Selection

The PRISMA flowchart (Figure 1) shows the process of identification and selection of published articles. Of the 3427 abstracts retrieved using our search strategy, 1553 were duplicates and, therefore, removed. Eight additional records were identified through citations in the reference lists of previous reviews and meta‐analyses, resulting in 1882 abstracts that underwent the screening process. A total of 1417 records were excluded based solely on title or abstract, whereas two could not be retrieved. The remaining 463 articles were thoroughly assessed, of which 428 were excluded due to the lack of clinical confirmation of ED (*n* = 202), the absence of a group diagnosed with a psychiatric condition (*n* = 71), and other reasons (for a comprehensive description, see Figure 1). Thirty‐five studies were included in the meta‐analysis. Moreover, we reported a descriptive synthesis of 22 studies that were considered ineligible for the meta‐analysis, and thus excluded, but worthy of a qualitative analysis. These studies were excluded because they reported (1) no activation coordinates (*n* = 1), (2) null findings (*n* = 7), (3) high‐level contrasts only (*n* = 12), or due to (4) other reasons (*n* = 2, including one mega‐analysis and one machine learning study).

### 3.2 Narrative Synthesis of Included Studies

Eligible studies, published between 2007 and 2023, investigated neural correlates of ED in different clinical populations, including BPD (*n* = 19), ADHD (*n* = 3), bipolar disorder (*n* = 3), major depressive disorder (*n* = 2), autism spectrum disorder (*n* = 2), panic disorder (*n* = 2), general anxiety disorder (*n* = 1), social anxiety disorder (*n* = 1), anorexia nervosa (*n* = 1), schizotypal personality disorder (*n* = 1), non‐suicidal self‐injury disorder (*n* = 1), PMDD (*n* = 1), SMD/DMDD (*n* = 1), and multiple psychiatric disorders (*n* = 1). Diagnoses were based on different editions of the DSM, including DSM‐III‐TR, DSM‐IV, DSM‐IV‐TR, and DSM‐5, as well as the ICD‐10. Sample sizes of the included studies ranged from 24 (*n* = 12 clinical patients, *n* = 12 HCs) to 130 (*n* = 62 clinical patients, *n* = 73 HCs), with a total sample size of 1989 (*n* = 1004 clinical patients, *n* = 985 HCs). Mean age ranged from 8.35 to 49.80 years for clinical patients, and from 7.74 to 35.84 years for HCs; *n* = 9 studies were conducted on youths, including children and/or adolescents, whereas *n* = 26 studies were conducted on adults only.

In 25 studies, clinical confirmation of ED was based on a significantly higher levels of symptoms in the patient group as compared to controls, as attested by questionnaires, structured or unstructured clinical interviews. In 10 other articles, instead, confirmation of ED for patients was based on standardized diagnostic criteria of specific psychiatric conditions, including *n* = 1 study on DMDD, *n* = 1 study on SMD, and *n* = 8 studies on BPD with explicitly confirmed DSM‐5 criteria 6 (“Affective instability due to a marked reactivity of mood”) and/or 8 (“Inappropriate, intense anger or difficulty controlling anger”). Different definitions of ED were provided by the included studies, of which the most frequently reported were affective instability (*n* = 7 studies) and affective lability (*n* = 6 studies); ED measures were substantially different across the included studies, and the most frequently used were the affective lability scale (Harvey et al. 1989) and the ER questionnaire (Gullone and Taffe 2012). Despite this operational heterogeneity, the constructs assessed across studies converged conceptually on a common dimension characterized by heightened emotional reactivity, rapid mood fluctuations, and impaired emotion modulation. Therefore, although clinical tools varied, they targeted shared components of disrupted affective processing in ED rather than distinct psychopathological manifestations.

As far as the recording of functional brain data is concerned, the majority of studies (*n* = 29 studies) employed a 3T MRI scanner, whereas the remaining (*n* = 6 studies) relied on a 1.5T machine. As expected, the experimental design varied substantially between studies, with the passive viewing of emotionally laden pictures being the most frequently employed (*n* = 15 studies). MRI images were processed using FSL, AFNI, BrainVoyager, and different versions of SPM; coordinates were reported in the MNI (*n* = 26 studies) or Talairach (*n* = 9 studies) spaces. Data extracted from the included studies are summarized in Table S1. We classified *hyper‐activations* as all coordinates from studies reporting higher BOLD activity in ED populations compared to HCs (i.e., ED patients > HCs). Similarly, *hypo‐activations* were classified as all coordinates from studies reporting lower BOLD activity in ED populations compared to HCs (i.e., ED patients < HCs). As not all studies reported significant results for both contrast directions, each ALE analysis included only the experiments providing coordinates for the corresponding effect (ED > HC or ED < HC).

### 3.3 Narrative Synthesis of Excluded Studies

Please refer to Supporting Information, for a narrative synthesis of excluded studies.

### 3.4 ALE Meta‐Analysis Findings

#### 3.4.1 Hyper‐Activations

**FIGURE 2:**
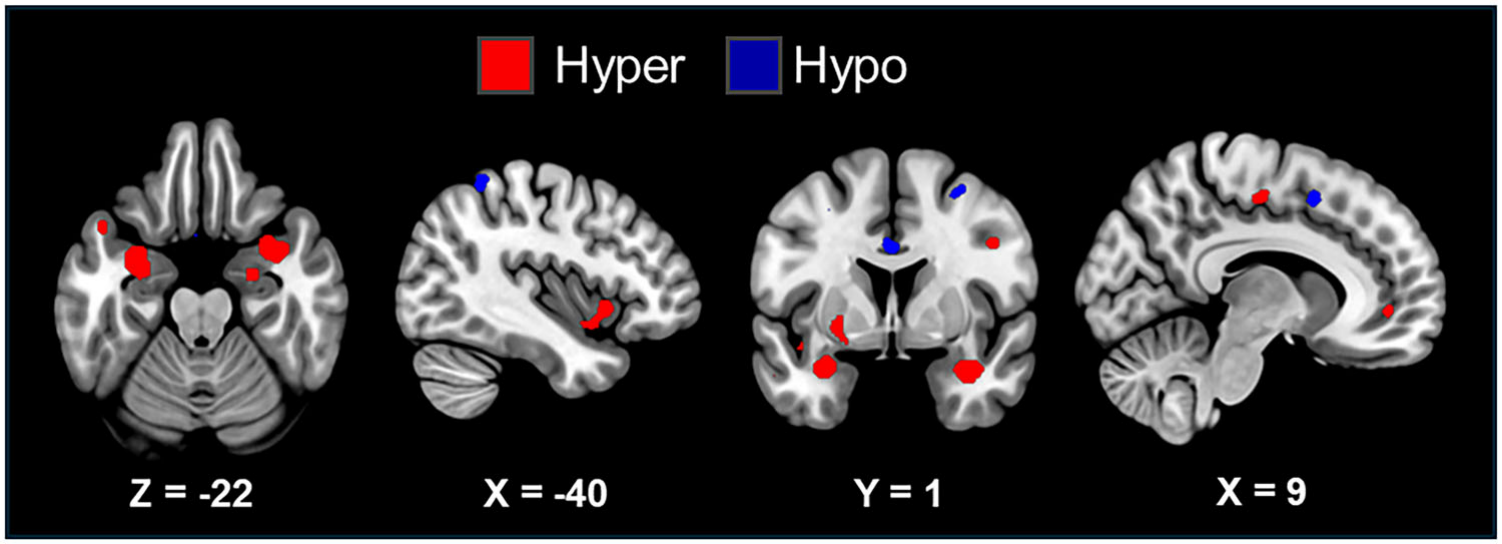
Hyper‐ and hypo‐activation clusters from canonical ALE and Bayesian thresholding.

Across 22 experiments (246 foci, 1118 subjects), we identified significant convergence in three clusters located in the bilateral amygdala and left insula. Using a minimum Bayesian thresholding approach, we detected five additional clusters beyond those identified through canonical ALE analysis. Although the locations of peak activations remained consistent in the Bayesian method, the clusters showed minor variations in size. These additional clusters were observed in the right precentral gyrus, right superior temporal gyrus, medial orbitofrontal gyrus, right supplementary motor area (SMA), and left putamen. Coordinates are reported in Table 1; see also Figure 2.

**TABLE 1:** Results from canonical activation likelihood estimation (ALE) and Bayesian thresholding.

Cluster	Brain region (local maxima)	Hemisphere	MNI			Cluster size (mm^3^)	mBF value	ALE value
			*X*	Y	Z			
ED > HC	Amygdala	L	−30	−4	−24	1514	805,655.6	0.026379
	Amygdala	R	36	2	−24	1421	800,922.2	0.026404
	Insula	L	−40	12	−6	871	6984.2	0.01931
	Precentral	R	46	0	32	286	5733.4	—
	Temporal superior	R	48	−40	14	304	2553.7	—
	M Orbito‐Frontal	R	12	44	−2	237	1267.5	—
	SMA	R	10	−16	50	375	914.6	—
	Putamen	L	−24	2	−4	270	718.3	—
ED < HC	SMA	R	4	10	50	519	8988.9	—
	Angular gyrus	R	32	54	42	326	1981.5	—
	Caudate	R	20	28	2	254	1378.7	—
	Middle occipital gyrus	R	42	−80	6	342	1053.6	—
	Anterior cingulum	L	−1	0	32	249	914	—
	Inf parietal lobule	L	−40	48	58	231	752.9	—

Abbreviations: ED, emotional dysregulation; HC, healthy control; mBF, minimum Bayes Factor; MNI, Montreal Neurological Institute; SMA, supplementary motor area.

#### 3.4.2 Hypo‐Activations

In 17 experiments (169 foci, 883 subjects), no significant hypo‐activation was found in ED populations compared to HCs using canonical ALE. However, applying the minimum Bayesian thresholding approach revealed six clusters: the right SMA, right angular gyrus (AG), right caudate, right middle occipital gyrus, left anterior cingulate cortex (ACC), and left inferior parietal gyrus. Coordinates are reported in Table 1; see also Figure 2.

#### 3.4.3 MACM Analysis

**FIGURE 3:**
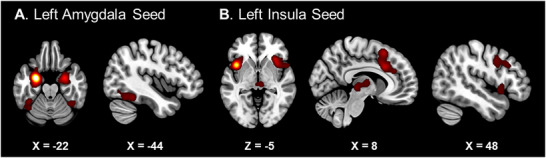
Coactivation clusters from MACM analysis A. Left Amygdala Seed B. Left Insula Seed.

For the right amygdala cluster, we identified 29 experiments involving 496 subjects and 364 foci, with no additional coactivations detected outside the seed region. For the left amygdala cluster, we identified 67 experiments with 1106 subjects and 1156 foci. Three clusters outside the seed were found, including the right amygdala and bilateral fusiform gyrus. For the left insula cluster, we identified 36 experiments with 636 subjects and 678 foci. Four clusters were observed outside the seed region, located in the bilateral cingulum, right insula, bilateral thalamus, and right middle frontal gyrus. Coordinates are reported in Table 2; see also Figure 3.

**TABLE 2:** Results from meta‐analytic connectivity modeling (MACM) analysis.

Cluster	Cluster size (mm^3^)	Brain region (local maxima)	Hemisphere	MNI			ALE value
				*X*	*Y*	*Z*	
Right amygdala seed	6480	Amygdala	R	32	2	−24	0.115679
		Insula	R	38	−4	−6	0.01452
Left amygdala seed	12,352	Amygdala	L	−26	−4	−22	0.2479488
		Globus pallidus	L	−16	2	−10	0.030122176
		Inferior frontal (pars orbitalis)	L	−36	22	−14	0.026731804
		Insula	L	−28	18	−16	0.022613104
		Globus pallidus	L	−12	8	−2	0.022159353
	6336	Amygdala	R	26	−4	−22	0.07918388
	3616	Fusiform	L	−42	−48	−20	0.041506976
		Fusiform	L	−42	−58	−18	0.033982128
		Fusiform	L	−46	−56	−16	0.03365106
		Fusiform	L	−46	−68	−10	0.02403146
	1256	Fusiform	R	40	−50	−22	0.028745998
Left insula seed	10,072	Middle cingulate	R	8	20	32	0.042422
		SMA	R	6	18	54	0.040711
		Anterior cingulate	R	6	36	24	0.022919
		Middle cingulate	L	−8	12	44	0.019847
		Anterior cingulate	L	−2	30	22	0.018174
	9904	Insula	L	−40	12	−6	0.147855
		Precentral	L	−52	8	6	0.02139
		Inferior frontal (pars opercularis)	L	−48	8	26	0.020908
		Insula	L	−44	14	16	0.016083
	8312	Insula	R	32	22	−4	0.043248
		Insula	R	36	16	−2	0.042831
		Precentral	R	56	14	2	0.025229
	5288	Thalamus	L	−4	−22	4	0.028294
		Thalamus	R	10	−8	4	0.025971
		Thalamus	R	6	−24	0	0.025398
	2424	Precentral	R	46	6	36	0.023942
		Middle frontal	R	50	22	30	0.022849
		Middle frontal	R	52	32	30	0.015246

Abbreviations: ALE, activation likelihood estimation; MNI, Montreal Neurological Institute; SMA, supplementary motor area.

### 3.5 Functional Decoding With NeuroSynth

**FIGURE 4:**
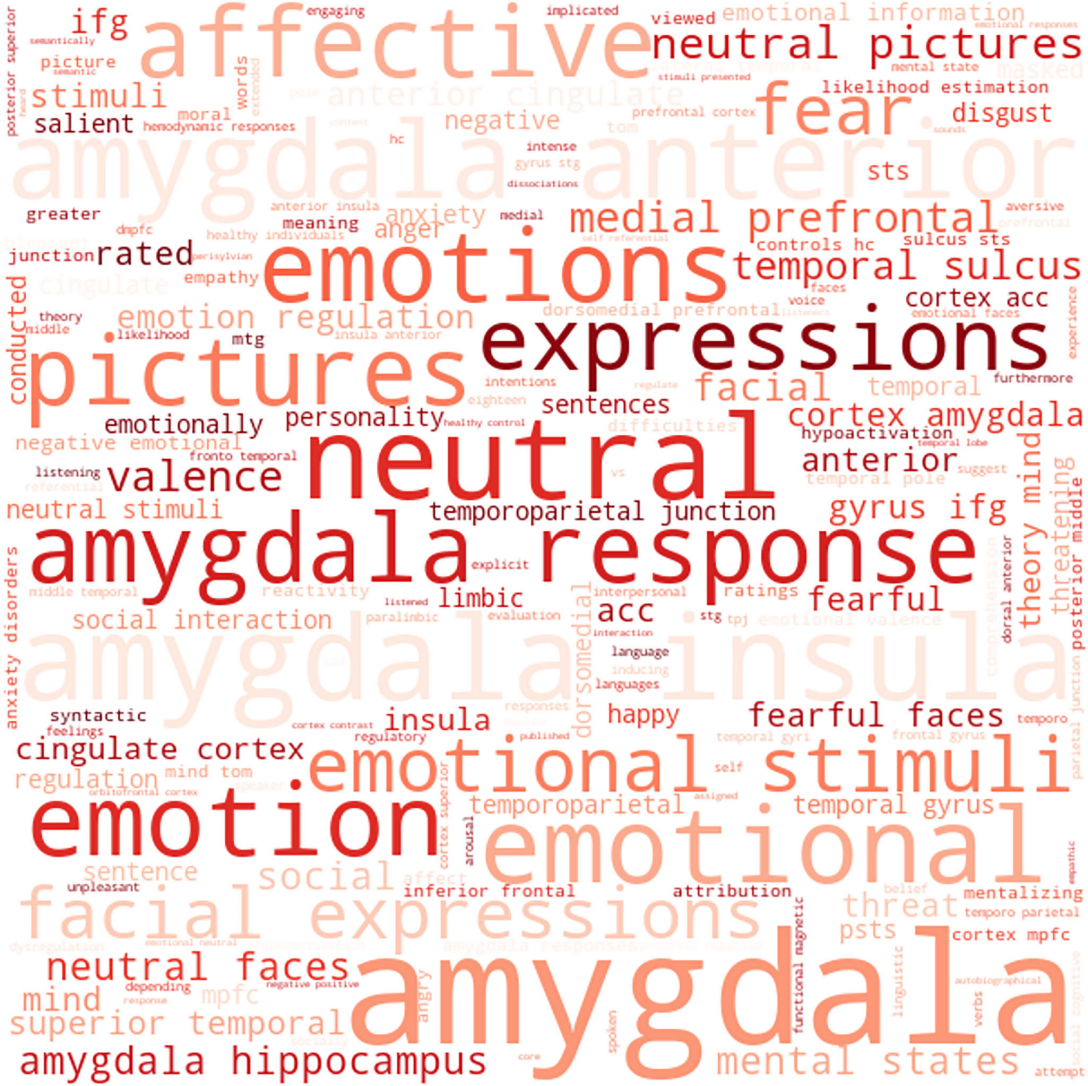
NeuroSynth word cloud related to hyper‐activation maps.

**FIGURE 5:**
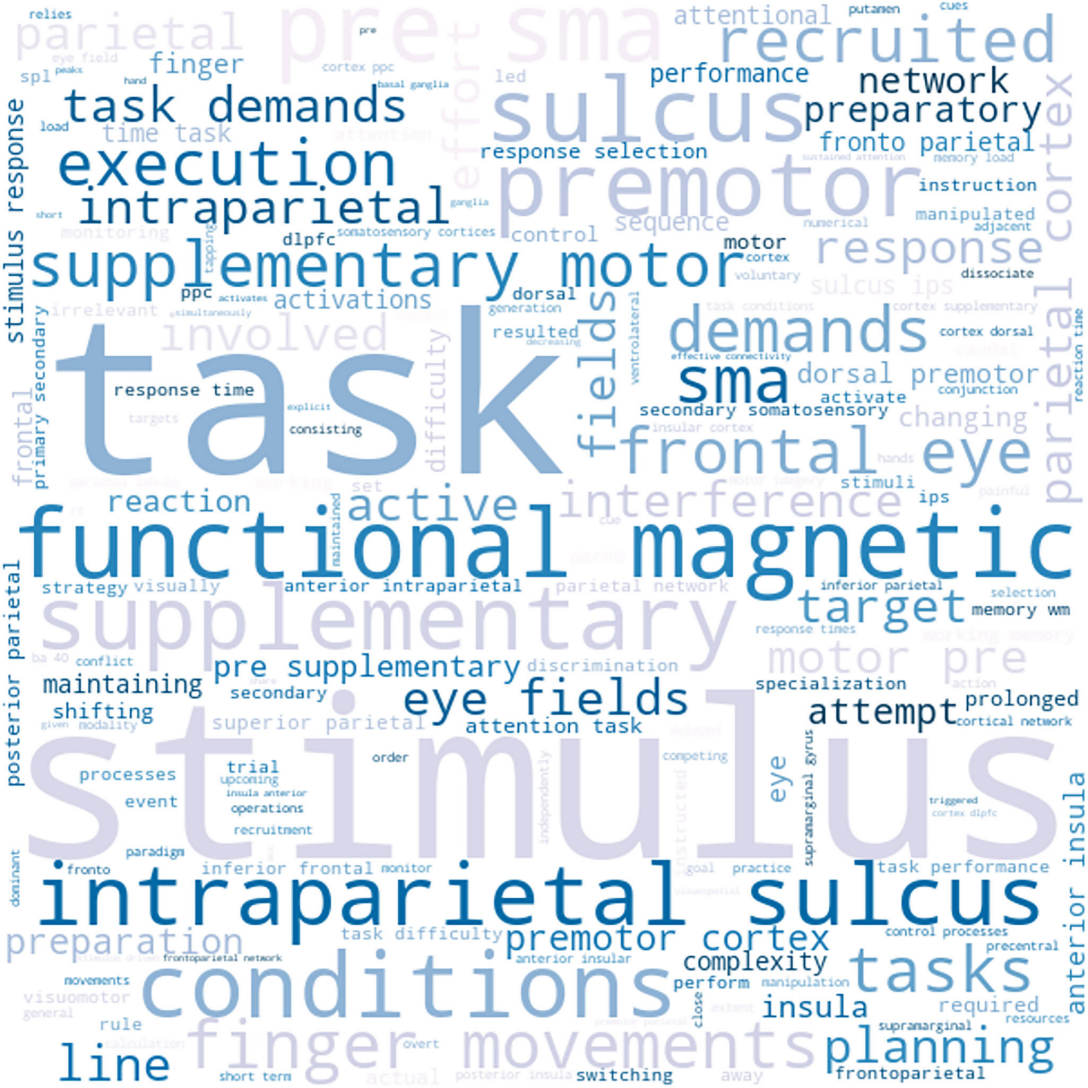
NeuroSynth word cloud related to hypo‐activation maps.

NeuroSynth decoding enables the association of whole‐brain activation maps derived from ALE analysis with terms frequently used in published fMRI studies, based on their Pearson's *r* correlations (Figures 4 and 5). Specifically, decoding the ALE‐based maps of hyper‐activation reveals that the terms with the highest correlations are mainly related to the affective and emotional domain (e.g., “emotion,” “emotional,” “affective,” “amygdala,” and “fear”) (Figure 4). Conversely, the terms most correlated with the ALE‐based maps of hypo‐activation include “stimulus,” “task,” “functional magnetic,” and “pre‐SMA,” which lack a clear association with a specific cognitive domain and, especially, the emotional and affective one (Figure 5).

## 4 Discussion

This meta‐analysis study provides a comprehensive investigation into the neural underpinnings of ED, a transdiagnostic dimension linked to different psychiatric and neurodevelopmental disorders. A crucial issue for our approach concerned the operational heterogeneity of ED definitions across included studies. Although different terms and clinical tools were used, our inclusion criteria focused on a shared dimensional construct centered on heightened emotional reactivity and regulatory impairment. From a coordinate‐based meta‐analytic perspective, neurobiological divergence across constructs would be expected to reduce spatial convergence. Instead, our results provided evidence for a robust convergence in regions known to be implicated in emotional salience and affective reactivity as well as regulatory regions, suggesting the presence of a shared neural core underlying different operational definitions of ED. By collecting data from 35 studies with a total of 1989 participants, we first used a canonical ALE approach to explore brain regions consistently activated or deactivated across a wide variety of clinical populations. Findings highlight both hyper‐activation and hypo‐activation in specific brain regions, revealing important insights into the neural circuitry of ED.

Importantly, in the present study, ED was conceptualized as a transdiagnostic dimension rather than a disorder‐specific construct. Accordingly, the neural pattern identified here should not be interpreted as uniquely related to specific conditions but rather as reflecting a shared neurofunctional substrate underlying dysregulated affect across diagnostic categories. The convergence of hyper‐activation in limbic regions together with reduced prefrontal engagement may index an imbalance between affective salience detection, interoceptive awareness, and regulatory monitoring that is particularly relevant for ED. At the same time, this configuration likely overlaps with broad neural signatures of negative affectivity in general psychopathology reported in previous transdiagnostic meta‐analyses. In this sense, our findings may capture a core affective‐regulatory imbalance that characterizes ED while also reflecting a common neural substrate of emotional disturbance across psychiatric conditions.

Hyper‐activations in emotion‐related brain regions, including the bilateral amygdala and the left insula, align with classical theories of ED, which emphasize heightened emotional reactivity to salient stimuli (Petrovic and Castellanos 2016). These regions are traditionally implicated in the processing of emotional stimuli, including threat detection and emotional salience, making them critical players in understanding ED (Fox et al. 2015; Gasquoine 2014). Particularly, hyper‐activation of the amygdala, a region deeply involved in emotional processing, is a consistent finding in affective disorders, including those marked by ED (Viering et al. 2022). The amygdala is central to the detection of emotional significance, particularly in threat‐related stimuli. In conditions such as BPD and ADHD, where emotional reactivity is often dysregulated, heightened amygdala activity reflects a potential failure in emotional regulation, leading to exaggerated emotional responses (e.g., anger, anxiety, or fear) to emotionally neutral or ambiguous stimuli (Kamphausen et al. 2013). This overactivity may contribute to the impulsivity and mood reactivity that characterize ED in these disorders (Masi et al. 2021). The insula, known to be involved in interoception and emotional awareness (Craig 2009), also shows hyper‐activation in ED populations. This suggests that individuals with ED may have an exaggerated awareness of their emotional state and internal condition, which could contribute to the intensity and lability of their emotional experiences (Atzil et al. 2023; Viering et al. 2021a). Additionally, the insula role in emotional processing (Klucken et al. 2012), particularly in relation to disgust and fear, underscores its relevance in disorders where emotional experiences are heightened and often difficult to regulate.

Incorporating the Bayesian thresholding approach further expanded these findings, identifying additional regions associated with hyper‐activation. These included areas like the precentral gyrus, the superior temporal gyrus, the medial orbitofrontal gyrus, and the putamen, which are known to be involved in motor control and sensory processing (Penfield and Boldrey 1937), decision‐making (Yoo and Hayden 2018), reward processing (Levy and Glimcher 2012), and emotional expression (Lettieri et al. 2019). Their inclusion suggests a broader network of emotional processing and emotion‐driven action initiation involved in ED, reinforcing the notion that ED is not only a disturbance of emotion processing confined to the amygdala and insula but may involve a more complex and integrated network of brain regions (Viering et al. 2021b). Within this network, the medial orbitofrontal gyrus, largely overlapping with the ventromedial prefrontal cortex, has been shown to store categorical representations of emotions that are independent of the sensory modality, whereas the superior temporal gyrus maps the valence dimension of emotions using an abstract code (Lettieri et al. 2024). Moreover, the involvement of these regions in patients with ED could contribute to the clinical expression of the condition through an impaired motoric expression of emotions and subsequent behavioral dysregulation in emotionally charged situations, such as impulsive behaviors or aggression (Giller et al. 2022); an impaired inhibitory control along with impulsive decision‐making under emotional stress, as well as a disrupted reward processing during emotional experiences (Adisetiyo and Gray 2017); and finally, an impaired ability to appropriately interpret and respond to social emotions or cues (Fantozzi et al. 2021; Flannery et al. 2016). Altogether, these findings suggest that individuals with ED may exhibit hyperactivity in regions responsible for an exaggerated action response to salient stimuli and difficulties in controlling behavioral impulses.

Although the hyper‐activation findings provide a clear picture of heightened emotional reactivity, the hypo‐activation results are more difficult to interpret. Notably, no significant hypo‐activation was found in ED populations using canonical ALE methods. However, the Bayesian thresholding approach revealed several regions with reduced activity, such as the right SMA, the right AG, and the left ACC. As these effects did not survive the standard cluster‐level FWE correction applied in the canonical ALE analysis, they should be interpreted with caution and cannot be considered as providing robust evidence of functional hypo‐activation. Particularly, ACC has long been implicated in ER, cognitive control, and decision‐making (Kar et al. 2013; Smith and Lane 2016), and its reduced activity in individuals with ED may reflect impairments in the cognitive control processes necessary to modulate emotional responses. The role of ACC in error detection and conflict monitoring (Swick and Turken 2002) suggests that hypo‐activity in this region could contribute to difficulties in inhibiting inappropriate emotional responses in exciting situations. The involvement of motor‐related regions like the SMA may reflect difficulties in executive control and action preparation during emotionally stressful situations (Chen et al. 2017). Impaired motor planning and action initiation could further exacerbate ED, especially in contexts where behavioral responses are required to manage emotions effectively, potentially explaining impulsive and dysregulated emotional outbursts. Last, the activity of AG is associated with higher order cognitive functions, including attention and social cognition (Kubit and Jack 2013; Lettieri et al. 2019; Seghier 2023); its hypo‐activation suggests that individuals with ED may experience difficulties in attentional control or perspective‐taking, which are noteworthily essential for regulating emotional reactions and understanding social cues. These findings suggest that the neural underpinnings of ED involve not only hyper‐activation in regions associated with emotional salience and impulsivity but also hypo‐activation in areas crucial for cognitive control, error monitoring, and the regulation of emotional responses, highlighting a broad disruption in the neural circuits responsible for ER (Petrovic and Castellanos 2016). However, these Bayesian‐derived clusters should be better interpreted as exploratory patterns that may guide future investigations rather than as definitive evidence of functional alterations in ED.

To further explore the functional relevance of the identified brain regions, we conducted a MACM analysis that assesses the coactivation patterns of identified clusters across a wide range of experiments, revealing how these regions interact during emotional processing. The MACM analysis provides, indeed, further insights into the functional connectivity of the identified regions. For example, the amygdala showed strong convergence within itself, with no additional coactivations detected outside the seed region, indicating that amygdala activity in individuals with ED is strongly self‐constrained. This is consistent with findings that heightened amygdala activation is isolated and overexpressed (Kirk et al. 2022), often not adequately regulated by higher‐order brain regions, such as the ACC, which typically exerts top‐down control over emotional responses. On the other hand, the left insula exhibited coactivation with regions involved in sensory processing and cognitive control, such as the bilateral thalamus, the bilateral cingulum, and the right middle frontal gyrus, suggesting that ED may involve a broader network of brain areas that link sensory input, emotional awareness, and regulatory control. This highlights the interoceptive and emotion‐focused nature of ED, pointing to a dysregulated feedback loop between sensory information and emotion processing.

The functional decoding analysis that we conducted using NeuroSynth further supports the conceptualization of ED as a failure in appropriate emotional processing and regulation. Hyper‐activation findings are strongly correlated with terms related to the emotional domain, reflecting the heightened emotional responses observed in ED. Conversely, the hypo‐activation of areas like the SMA and AG is linked to more cognitive and task‐oriented terms, reinforcing the idea that ED involves not only emotional processing but also impairments in higher level processes like attention, cognitive flexibility, and decision‐making (Petrovic and Castellanos 2016). These findings suggest that ED is not merely a matter of heightened emotional responses but involves a failure of regulation across both emotional and cognitive domains (Petrovic and Castellanos 2016). Individuals with ED may struggle not only with intense emotions but also with the cognitive control necessary to modulate these emotions effectively in different contexts.

The regions identified through our meta‐analysis hold important implications for understanding the neural mechanisms underlying ED in specific clinical populations. Disorders such as BPD, ADHD, and bipolar disorder (BD), which are strongly associated with ED, may share common neurobiological pathways that contribute to their characteristic emotional instability (Petrovic and Castellanos 2016). The consistent limbic findings could inform therapeutic strategies targeting emotion‐focused interventions (Iskric and Barkley‐Levenson 2021). Instead, hyper‐activations in other regions suggest a way through which treatments addressing not only cognitive and attentional deficits but also impulsivity and reduced behavioral control, which often overlap with ED, may be effective (Lenzi et al. 2017).

Despite the valuable insights gained from this meta‐analysis, several limitations should be acknowledged. First, the cross‐sectional and correlational nature of brain imaging studies included in this meta‐analysis precludes causal inferences about the brain activity patterns associated with ED. Second, the variability in experimental paradigms, which translates into different stimuli, task designs, and participant demographics, may have introduced heterogeneity in our analysis. In this perspective, the predominance of passive emotional viewing paradigms represents an important issue. Such tasks primarily probe bottom‐up affective salience processing and may therefore preferentially reveal limbic hyper‐reactivity. In contrast, explicit regulation paradigms engage frontoparietal control systems in a more task‐ and strategy‐dependent manner, which may reduce spatial convergence across studies. The absence of canonical hypo‐activation findings may thus reflect dispersion of regulatory effects across tasks rather than absence of regulatory dysfunction per se.

Moreover, the inclusion of a majority of studies focused on BPD patients could be considered a further limitation, as the overrepresentation of studies on this specific population may have skewed the findings, making them less generalizable to other clinical populations with ED, such as those with ADHD, mood disorders, or anxiety disorders. As a result, our findings might reflect patterns that are more specific to BPD and less representative of the broader spectrum of ED in other disorders, or, in other words, they may capture instability‐based ED rather than other forms, such as chronic irritability. However, similar limbic hyper‐responsivity has been documented across ADHD, BD, and mood disorders, suggesting that the observed convergence likely reflects shared neurofunctional variance rather than disorder‐specific effects. Nevertheless, future task‐stratified meta‐analysis with balanced diagnostic representation is warranted to disentangle subtype‐specific contributions. Moreover, to improve generalizability, future studies should employ balanced task paradigms across multiple diagnostic groups or even adopt dimensional recruitment strategies based on continuous ED severity rather than categorical diagnoses.

Additionally, the inclusion of studies employing heterogeneous operational ED definitions represents a major limitation of our transdiagnostic approach, as measurement variability may have introduced noise and reduced construct specificity. Thus, the findings of the present study should be interpreted as likely reflecting a shared neurofunctional substrate of broad ED rather than disorder‐specific or symptom‐specific neural signatures. In this sense, heterogeneity at the measurement level does not necessarily imply neurobiological heterogeneity. Finally, although the ALE and MACM techniques provide robust evidence of region‐specific activations, they are dependent on the quality and scope of the included studies, which may introduce publication bias or underrepresentation of certain brain regions.

This meta‐analysis provides a detailed examination of the neural circuits involved in ED, revealing a heightened sensitivity of the amygdala and the insular cortex, two regions traditionally involved in emotional processing, as a marker of the transdiagnostic phenomenon of ED. At the same time, through the Bayesian statistics, we also reveal that other brain regions associated with processes of a more cognitive nature, such as the ACC and the AG, also demonstrate a specific altered response in patients with ED across several psychiatric diagnoses. These findings emphasize the complex, transdiagnostic nature of ED and point toward the need for a multidimensional approach that addresses both the emotional reactivity and the cognitive control deficits observed in affected individuals. Future research should further clarify the causal relationships between these brain regions and ED. Longitudinal studies exploring the development of ED in different populations, as well as studies including neurofunctional patterns as intervention outcomes, will be crucial for developing targeted, effective treatments. Additionally, incorporating more diverse clinical populations and task‐based paradigms may help refine our understanding of the neural basis of ED across a wider array of psychiatric and neurodevelopmental disorders.

## Author Contributions

**Riccardo Loconte**: methodology, investigation, data curation. **Gianluca Sesso**: conceptualization, investigation, writing – original draft, methodology, validation, visualization, writing – review and editing, formal analysis, supervision. **Ern Wong**: conceptualization, methodology, formal analysis, software. **Francesca Terigi**: methodology, investigation, data curation. **Elisa Giani**: methodology, investigation, data curation. **Matteo Bucci**: methodology, investigation, data curation. **Maurilio Menduni De Rossi**: methodology, investigation, data curation. **Davide Coraci**: sofware. **Annarita Milone**: supervision, validation. **Gabriele Masi**: supervision, validation. **Luca Cecchetti**: supervision, validation, writing – review and editing.

## Funding

This work has been partially supported by Italian Ministry of Health (Ricerca Corrente 2024 and the “5 × 1000” voluntary contributions).

## Ethics Statement

The authors have nothing to report.

## Consent

The authors have nothing to report.

## Conflicts of Interest

Dr. Gabriele Masi has received research grants from Lundbeck and Humana, was in an advisory board for Angelini, and has been speaker for Angelini, FB Health, Janssen, Lundbeck, and Otsuka. All the other authors declare no conflicts of interest.

## Supporting information




**Supplementary TableS1**: brb371376‐sup‐0001‐TableS1.xlsx

**Supplementary SuppMat**: brb371376‐sup‐0002‐SuppMat.rar

**Supplementary SuppMat**: brb371376‐sup‐0003‐SuppMat.zip

**Supplementary SuppMat**: brb371376‐sup‐0004‐SuppMat.docx

## Data Availability

The data that support the findings of this study are available from the corresponding author upon reasonable request.
