# Supplementary material for: Neurofunctional Correlates of Emotional Dysregulation: Systematic Review and ALE Meta‐Analysis
Source: Brain Behav. 2026 Apr 16;16(4):e71376. doi: 10.1002/brb3.71376 (PMC13087441; doi:10.1002/brb3.71376)
Supplement: Supplementary file 4 — Supplementary SuppMat: brb371376‐sup‐0004‐SuppMat.docx [file BRB3-16-e71376-s001.docx]

1. **Clinical measures for the assessment of ED**

Here below we report a comprehensive list of the available clinical measures used for the assessment of ER/ED in clinical and research setting:

- Abbreviated Dysregulation Inventory
- Adolescent Emotion Regulation Questionnaire
- Adolescent Quality of Life Mental Health Scale – Emotion Regulation subscale
- Affect Dysregulation Scale
- Affect Regulation and Experience Q-sort-Questionnaire Version (AREQ-QV)
- Affective Control Scale (ACS)
- Affective Intensity Measure (AIM)
- Affective Lability Scale (ALS)
- Affective Lability Scale Short Form (ALS-S)
- Affective Reactivity Index (ARI)
- Anger Response Inventory
- Anger Rumination Scale
- Assessment Profile of Disruptive Behavior (MAP-DB) – Temper Loss subscale
- Auxiliary subscale of the Centre for Neurologic Study-Lability Scale (CNS-LS)
- Barkley Current Behavior Scale
- Barkley Deficits in Executive Functioning Scale (BDEFS)
- Barkley Emotional Dysregulation Scale
- Behavior Rating Inventory of Executive Function (BRIEF)
- Brown ADD Rating Scales for Children, Adolescents and Adults (BAADS)
- Child and Adolescent Flexible Expressiveness Scale
- Child Behavior Checklist – Dysregulation Profile (CBCL-DP) or EDI scale
- Children Affective Lability Scale
- Cognitive Emotion Regulation Questionnaire (CERQ)
- Cognitive Emotion Regulation Questionnaire for Children
- Conners Parent Rating Scale
- Conners’ Adult ADHD Rating Scale (CAARS) – Impulsivity/Emotional Lability Scale (I/ELS)
- Conners’ Global Index – Emotional Lability Scale (CGI)
- Conners’ Rating Scales Revised – Emotional Lability (CRS-R)
- Cyclothymic Hypersensitive Temperament (CHT)
- Deficient emotional self-regulation scale (DESRS)
- Difficulties in Emotion Regulation Scale (DERS)
- Distress Tolerance Scale (DTS)
- Ecological Momentary Assessment (EMA)
- Emotion Dysregulation Inventory (EDI)
- Emotion Dysregulation Measure (EDM)
- Emotion Dysregulation Scale (EDS)
- Emotion Expression Scale for Children
- Emotion Reactivity Scale
- Emotion Regulation Ability Scale
- Emotion Regulation and Social Skills Questionnaire
- Emotion Regulation Checklist (ERC)
- Emotion Regulation Index for Adults and Children (ERICA)
- Emotion Regulation Index for Children and Adolescents
- Emotion Regulation Interview (ERI)
- Emotion Regulation Questionnaire (ERQ)
- Emotion Regulation Scale (ERS)
- Emotionality Activity Sociability and Impulsivity (EASI)
- Expression and Emotion Scale for Children (EESC)
- Frustration Discomfort Scale (FDS)
- General Emotion Dysregulation Measure (GEDM)
- Life Problems Inventory – Emotional Dysregulation
- Mood Lability Scale (MLS)
- Negative Mood Regulation Scale
- Reactivity, Intensity, Stability and Polarity (RIPoSt) scale
- Regulation of Emotions Questionnaire
- Resiliency Scales in Children and Adolescents (RSCA)
- Responses to Emotions Questionnaire (REQ)
- Self-Regulation Scale
- State Difficulties in Emotion Regulation Scale (SDERS)
- Strategies of Anger Regulation for Adolescents
- Strengths and Difficulties Questionnaire (SDQ) – Dysregulation Profile
- Temperament Evaluation of the Memphis, Pisa, Paris and San Diego Questionnaire (TEMPS-A)
- Wender–Reimherr Adult Attention Deficit Disorder Scale (WRAADDS)
- Youth Self Report – EDI scale or Dysregulation Profile

1. **Narrative synthesis of excluded studies**

One study did not report coordinates of peak activations during the fMRI task. This study (1) aimed to investigate the hemodynamic correlates of passive viewing and voluntary downregulation of negative emotions by means of the reappraisal strategy detachment in 36 adolescent female patients with Anorexia Nervosa (AN) compared to pairwise age-matched healthy controls. Clinically, patients reported equal use of the emotion regulation strategy suppression according to the Emotion Regulation Questionnaire but significantly less use of the reappraisal strategy reinterpretation compared to healthy controls. The authors first revealed an altered hemodynamic activity in the bilateral amygdala as well as in the dorsolateral Prefrontal Cortex (dlPFC) in both groups during the emotion induction and regulation tasks. Hence, a ROI-based analysis was further performed showing an increased activity in the right amygdala and bilateral dlPFC in response to passively viewing negative pictures in AN patients compared to controls, while no group differences were visible in either ROI during explicit emotion regulation. Finally, whole-brain exploratory analyses to assess the main effect of group were also conducted with no reported coordinates of peak activations.

Seven studies reported null findings and could not be included in the meta-analysis. Stoddard et al. 2017 (2) focused on the shared and unique neural correlates of irritability and anxiety in youths, highlighting their effects on amygdala connectivity during the processing of facial emotions. This study involved a cross-sectional fMRI analysis of 115 youths, revealing that irritability and anxiety jointly influenced left amygdala connectivity to the medial prefrontal cortex. Specifically, heightened levels of both traits were linked to decreased connectivity during the viewing of angry faces. Instead, task-related BOLD signal variations across groups led no significant findings. This suggests that studying irritability and anxiety in isolation may overlook important neurobiological insights, emphasizing the need for a more integrated approach to understanding ED in youth.

Kircanski et al. 2018 (3) further dissected the neural mechanisms underlying co-occurring symptoms of irritability and anxiety during threat orientation tasks. Utilizing a bifactor analysis, the study identified unique and shared neural correlates among 197 young participants. Results indicated that while irritability was dimensionally linked to increased activity in areas such as the insula and prefrontal cortex, anxiety was dimensionally associated with decreased amygdala connectivity; no significant results were reported from group comparisons. These findings illustrate a double dissociation in the neural pathways associated with these dimensional traits, suggesting that traditional diagnostic categories may hide significant differences in underlying neural processes.

Similarly, Tseng et al. 2019 (4) examined the age-specific neural correlates of irritability in youths using a frustration fMRI paradigm. The study revealed that irritability was positively associated with activation in frontal-striatal regions during tasks involving attention orienting following frustration. Notably, younger participants displayed a stronger relationship between irritability and neural activation, suggesting developmental factors influence emotional dysregulation and neural responses in youth. Null findings were reported from group analysis.

Denny et al. 2018 (5) investigated neural correlates of ED in BPD, focusing on the salience network activity in response to negative stimuli. The study found that BPD adult patients exhibited an increased activity over the salience network upon reencountering negative images, indicating a lack of habituation compared to control groups, whereas no group differences were detected using a voxel-wise approach. This sensitization predicted greater self-reported negative affect, highlighting a potential neurocognitive target for interventions aimed at regulating emotional responses in BPD.

In examining NSSI behaviors, Mayo et al. 2021 (6) investigated emotional reactivity in 30 adolescents with NSSI. Their findings indicated enhanced emotional reactivity linked to anterior insula activity, despite no differences in self-reported affect nor in group comparisons of voxel-wise BOLD signal activity. This dissociation points to potential suppression of emotional experiences in adolescents with NSSI, suggesting new avenues for therapeutic interventions focused on emotional awareness and expression.

Lamers et al. 2021 (7) delved into the neural activation patterns of patients with BPD when processing happy faces, revealing hyperactivation in the caudate compared to healthy controls. While BPD women reported less acceptance of positive emotions, no correlation with neural activation was found. Moreover, a voxel-wise approach led to null findings. This suggests that emotional acceptance might be disrupted in BPD, potentially influencing how positive stimuli are processed.

Lastly, Carvalho Fernando et al. 2023 (9) examined emotion regulation strategies in BPD women. Their study found that individuals with BPD showed altered neural activity patterns, with under-activation in the insula during emotion acceptance tasks and overactivation in the caudate during suppression tasks. Group differences in voxel-wise neural activations were not significant. This reflects the habitual reliance on suppression as a coping mechanism, highlighting the need for interventions that promote healthy emotional processing.

Twelve studies performed only higher order interactions, and the great majority was conducted by the same research group led by Ellen Leibenluft. The study by Adleman and colleagues (11) investigates the neurophysiological underpinnings of cognitive flexibility deficits in children with SMD and pediatric BD compared to healthy volunteers. Using fMRI during a response reversal task, the researchers focused on brain regions including the caudate, inferior frontal gyrus, and cingulate gyrus. Results indicated that both SMD and BD participants exhibited reduced activation in the caudate compared to healthy youth when responding to errors. Notably, the IFG activation patterns differed, with SMD participants showing a more significant deficit than those with BD. Exploratory analyses revealed additional involved regions, such as the superior parietal lobule and inferior temporal gyrus, that differentiated the two clinical groups. These findings suggest that while there are shared neural perturbations related to error responses in both disorders, distinct cognitive flexibility deficits in the IFG may characterize SMD.

Thomas et al. 2012 (12) compared the neural responses to facial emotions among youth with BD and SMD to healthy volunteers. Participants underwent fMRI while rating morphed facial expressions transitioning from neutral to angry or happy. The results revealed that healthy controls exhibited a positive correlation between amygdala activity and the perception of anger, a response absent in both patient groups. Interestingly, SMD showed increased parietal and frontal activity in response to happy faces, while BD demonstrated decreased activity in these regions. These findings highlight that while both BD and SMD exhibit impairments in processing emotional faces compared to healthy controls, they also show distinct neural modulation patterns in response to emotional stimuli, indicating differences in their underlying neurophysiology.

Niedtfeld and coworkers in 2012 (13) explored how pain influences emotional regulation in individuals with BPD through functional connectivity analysis. Patients with BPD were examined during an fMRI task involving negative emotional stimuli followed by painful stimulation. Results showed that BPD patients had enhanced negative coupling between limbic regions and prefrontal areas when experiencing pain alongside emotional arousal. Conversely, positive connectivity was observed when neutral images were paired with painful sensations, suggesting that pain may help modulate limbic activity. These findings offer new insights into the neural mechanisms of affect regulation in BPD, emphasizing how pain might serve as a maladaptive emotional regulation strategy.

Thomas et al. 2013 (12) investigated amygdala dysfunction in youth with chronic irritability as SMD and pediatric BD during an implicit emotion processing task featuring various facial expressions. Both groups displayed increased right amygdala activity compared to healthy volunteers, indicating shared dysfunction. However, they diverged in activation patterns in regions such as the posterior cingulate and inferior parietal lobe, where SMD showed deactivation to fearful faces while BD did so to angry faces. These results underscore that while both disorders exhibit similar amygdala dysfunctions, they also display distinct abnormalities in brain regions involved in processing emotional information.

The same authors in 2014 (14) examined how youth with BD and SMD process emotional faces, both with and without awareness, during an fMRI task. The analysis revealed that BD and SMD groups exhibited heightened neural activity during non-aware processing of emotional faces compared to healthy volunteers, who showed the opposite pattern. Notably, both patient groups displayed different activation patterns in brain regions associated with face processing and emotional regulation. These findings suggest that awareness is not necessary for recognizing emotional faces and that distinct neural mechanisms underlie mood disorders, highlighting the complexity of face emotion labeling deficits in BD and SMD.

Tseng and colleagues in 2016 (15) replicated previous findings on neural responses to masked and unmasked emotional face processing in youth with SMD. Using fMRI, the study found that SMD participants exhibited increased activation in regions like the para-hippocampal gyrus and superior temporal gyrus when processing angry faces, while showing decreased activation in areas like the insula when processing happy faces. Overall, SMD youth demonstrated heightened ventromedial prefrontal cortex activation during masked emotion processing. These results suggest that SMD individuals have altered sensitivity to negative and positive emotions, potentially contributing to symptoms such as irritability and aggression.

Wiggins et al. 2016 (16) investigated the neural mechanisms underlying irritability in youth with DMDD and BD through an fMRI face emotion labeling task. Findings revealed that while both groups exhibited similar levels of irritability and labeling accuracy, their neural correlates differed. In DMDD, irritability was consistently correlated with amygdala activity across emotional intensities, whereas in BD, this correlation was only evident for fearful faces. Moreover, the DMDD group showed stronger neural associations with ambiguous emotional stimuli. These results suggest diagnostic specificity in the neural underpinnings of irritability across these mood disorders, indicating the need for tailored therapeutic approaches.

Niedtfeld et al 2017 (17) conducted a longitudinal study examining the impact of DBT on pain-mediated affect regulation in individuals with BPD. In the cross-sectional study at baseline before DBT, participants underwent fMRI while experiencing emotional stimuli and painful heat. Initially, BPD patients displayed altered amygdala responses and connectivity patterns during painful stimuli, indicative of using pain for emotional regulation. Post-therapy, these neural alterations diminished, suggesting that DBT effectively alters the maladaptive use of pain for emotion regulation. This study highlights the potential of therapeutic interventions in modifying neural mechanisms underlying emotional dysregulation in BPD.

Pagliaccio and coworkers in 2017 (18) explored the attentional deficits in youth with DMDD and ADHD through fMRI. Participants performed an attention task while their brain activity was monitored. Results showed that DMDD youths exhibited distinct patterns of neural activity related to reaction time compared to healthy controls, particularly in regions like the paracentral lobule and superior parietal lobule. While both DMDD and ADHD groups displayed similar blunted compensatory neural responses during long reaction times, DMDD youths also demonstrated increased reaction time variability. This study identifies both unique and shared neural signatures of attentional processes in DMDD and ADHD.

Wiggins et al. 2017 (19) examined the neural markers associated with familial risk for BD in youths during a face emotion labeling task. The study categorizes participants into three groups: those with BD, high-risk youth with a family history of BD, and low-risk youth. Results indicated shared deficits in higher-order face processing regions among BD and high-risk participants, suggesting potential risk endophenotypes. Additionally, unique neural patterns were identified, pointing to resilience markers in high-risk youths and disorder-related alterations in those with BD. These findings suggest the potential for using neuroimaging to identify at-risk individuals and develop targeted preventive interventions.

Zhang et al 2021 (20) investigated the interplay between irritability and callous-unemotional (CU) traits in adolescents during a looming threat task, using fMRI. The results indicated that higher irritability was linked to increased brain activity in response to looming threats, particularly in those with low CU traits. Conversely, higher CU traits were associated with reduced threat responsiveness, especially in highly irritable youth. These findings highlight the complex neurobiological interactions between irritability and CU traits, suggesting that CU traits may dampen the threat responsiveness typically associated with irritability.

The same research group in 2023 (21) examined how adolescents with conduct disorder (CD) respond to rewards and punishments during an fMRI passive avoidance task. The findings reveal that CD adolescents exhibited reduced sensitivity to reward versus punishment compared to typically developing peers. Notably, the severity of callous-unemotional (CU) traits was associated with this diminished responsiveness, while irritability did not show a significant relationship. This suggests that the dysfunction in reinforcement-based decision-making in CD is more closely tied to CU traits, emphasizing the need to consider different affective components when assessing and treating CD.

Finally, two additional studies were excluded for other reasons than those reported so far. One study (8) performed a mega-analysis on individual participant data coming from three different fMRI studies including, overall, a total sample of 192 women with ED – 49 with BPD, 62 with PTSD and 81 healthy controls. Across the three studies, participants were shown pictures with emotional contents and a significantly different expression of the neural signatures between neutral and negative pictures strongly emerged as expected. Nonetheless, no differences in the hemodynamic response to pictures were observed between participants with and without ED, thus raising relevant issue concerning the neural signature related to individual differences in ED.

On the contrary, a preliminary fMRI study (10) followed a machine learning approach to examine the ability of the neural activity during a frustrating cognitive flexibility task to predict levels of irritability in a transdiagnostic sample of 69 youths with DMDD, ADHD, anxiety disorders and healthy controls. The parent and child reports of the Affective Reactivity Index (ARI) were used as dimensional measures of irritability. The connectome-based predictive modeling based on a machine learning approach was shown to predict child-reported irritability even adjusting for age, sex, medications, motion, ADHD, and anxiety symptoms. The predictive neural features were primarily located within sensorimotor, subcortical, and salience networks, as well as between these networks and frontoparietal and medial frontal networks. This preliminary evidence illustrates that, while more canonical approaches to fMRI data is not able to capture the neural signature of ED (8), machine learning modeling could overcome, at least in youth, such limitation.

**References**

1. Seidel M, King JA, Ritschel F, Boehm I, Geisler D, Bernardoni F, *et al.* (2018): Processing and regulation of negative emotions in anorexia nervosa: An fMRI study. *NeuroImage Clin* 18: 1–8.

2. Stoddard J, Tseng W-L, Kim P, Chen G, Yi J, Donahue L, *et al.* (2017): Association of Irritability and Anxiety With the Neural Mechanisms of Implicit Face Emotion Processing in Youths With Psychopathology. *JAMA psychiatry* 74: 95–103.

3. Kircanski K, White LK, Tseng W-L, Wiggins JL, Frank HR, Sequeira S, *et al.* (2018): A Latent Variable Approach to Differentiating Neural Mechanisms of Irritability and Anxiety in Youth. *JAMA psychiatry* 75: 631–639.

4. Tseng W-L, Deveney CM, Stoddard J, Kircanski K, Frackman AE, Yi JY, *et al.* (2019): Brain Mechanisms of Attention Orienting Following Frustration: Associations With Irritability and Age in Youths. *Am J Psychiatry* 176: 67–76.

5. Denny BT, Fan J, Fels S, Galitzer H, Schiller D, Koenigsberg HW (2018): Sensitization of the Neural Salience Network to Repeated Emotional Stimuli Following Initial Habituation in Patients With Borderline Personality Disorder. *Am J Psychiatry* 175: 657–664.

6. Mayo LM, Perini I, Gustafsson PA, Hamilton JP, Kämpe R, Heilig M, Zetterqvist M (2021): Psychophysiological and Neural Support for Enhanced Emotional Reactivity in Female Adolescents With Nonsuicidal Self-injury. *Biol psychiatry Cogn Neurosci neuroimaging* 6: 682–691.

7. Lamers A, Toepper M, Fernando SC, Schlosser N, Lenz E, Woermann F, *et al.* (2021): Caudate hyperactivation during the processing of happy faces in borderline personality disorder. *Neuropsychologia* 163. https://doi.org/10.1016/j.neuropsychologia.2021.108086

8. Sicorello M, Herzog J, Wager TD, Ende G, Müller-Engelmann M, Herpertz SC, *et al.* (2021): Affective neural signatures do not distinguish women with emotion dysregulation from healthy controls: A mega-analysis across three task-based fMRI studies. *Neuroimage: Reports* 1. https://doi.org/10.1016/j.ynirp.2021.100019

9. Fernando SC, Beblo T, Lamers A, Schlosser N, Woermann FG, Driessen M, Toepper M (2023): Neural correlates of emotion acceptance and suppression in borderline personality disorder. *Front PSYCHIATRY* 13. https://doi.org/10.3389/fpsyt.2022.1066218

10. Scheinost D, Dadashkarimi J, Finn ES, Wambach CG, MacGillivray C, Roule AL, *et al.* (2021): Functional connectivity during frustration: a preliminary study of predictive modeling of irritability in youth. *Neuropsychopharmacology* 46: 1300–1306.

11. Adleman NE, Kayser R, Dickstein D, Blair RJR, Pine D, Leibenluft E (2011): Neural correlates of reversal learning in severe mood dysregulation and pediatric bipolar disorder. *J Am Acad Child Adolesc Psychiatry* 50: 1173-1185.e2.

12. Thomas LA, Kim P, Bones BL, Hinton KE, Milch HS, Reynolds RC, *et al.* (2013): Elevated amygdala responses to emotional faces in youths with chronic irritability or bipolar disorder. *NeuroImage Clin* 2: 637–645.

13. Niedtfeld I, Kirsch P, Schulze L, Herpertz SC, Bohus M, Schmahl C (2012): Functional connectivity of pain-mediated affect regulation in Borderline Personality Disorder. *PLoS One* 7: e33293.

14. Thomas LA, Brotman MA, Bones BL, Chen G, Rosen BH, Pine DS, Leibenluft E (2014): Neural circuitry of masked emotional face processing in youth with bipolar disorder, severe mood dysregulation, and healthy volunteers. *Dev Cogn Neurosci* 8: 110–120.

15. Tseng W-L, Thomas LA, Harkins E, Pine DS, Leibenluft E, Brotman MA (2016): Neural correlates of masked and unmasked face emotion processing in youth with severe mood dysregulation. *Soc Cogn Affect Neurosci* 11: 78–88.

16. Wiggins JL, Brotman MA, Adleman NE, Kim P, Oakes AH, Reynolds RC, *et al.* (2016): Neural Correlates of Irritability in Disruptive Mood Dysregulation and Bipolar Disorders. *Am J Psychiatry* 173: 722–730.

17. Niedtfeld I, Schmitt R, Winter D, Bohus M, Schmahl C, Herpertz SC (2017): Pain-mediated affect regulation is reduced after dialectical behavior therapy in borderline personality disorder: a longitudinal fMRI study. *Soc Cogn Affect Neurosci* 12: 739–747.

18. Pagliaccio D, Wiggins JL, Adleman NE, Curhan A, Zhang S, Towbin KE, *et al.* (2017): Behavioral and Neural Sustained Attention Deficits in Disruptive Mood Dysregulation Disorder and Attention-Deficit/Hyperactivity Disorder. *J Am Acad Child Adolesc Psychiatry* 56: 426–435.

19. Wiggins JL, Brotman MA, Adleman NE, Kim P, Wambach CG, Reynolds RC, *et al.* (2017): Neural Markers in Pediatric Bipolar Disorder and Familial Risk for Bipolar Disorder. *J Am Acad Child Adolesc Psychiatry* 56: 67–78.

20. Zhang R, Bashford-Largo J, Lukoff J, Elowsky J, Carollo E, Schwartz A, *et al.* (2021): Callous-Unemotional Traits Moderate the Relationship Between Irritability and Threatening Responding. *Front psychiatry* 12: 617052.

21. Zhang R, Aloi J, Bajaj S, Bashford-Largo J, Lukoff J, Schwartz A, *et al.* (2023): Dysfunction in differential reward-punishment responsiveness in conduct disorder relates to severity of callous-unemotional traits but not irritability. *Psychol Med* 53: 1870–1880.
